# Structural analysis of health-relevant policy-making information exchange networks in Canada

**DOI:** 10.1186/s13012-017-0642-4

**Published:** 2017-09-20

**Authors:** Damien Contandriopoulos, François Benoît, Denise Bryant-Lukosius, Annie Carrier, Nancy Carter, Raisa Deber, Arnaud Duhoux, Trisha Greenhalgh, Catherine Larouche, Bernard-Simon Leclerc, Adrian Levy, Ruth Martin-Misener, Katerina Maximova, Kimberlyn McGrail, Candace Nykiforuk, Noralou Roos, Robert Schwartz, Thomas W. Valente, Sabrina Wong, Evert Lindquist, Carolyn Pullen, Anne Lardeux, Melanie Perroux

**Affiliations:** 1 Faculté des Sciences Infirmières, Université de Montréal, C.P. 6128 succursale Centre-ville, Montréal, QC H3C 3J7 Canada; 2 National Collaborating Centre for Healthy Public Policy (NCCHPP), 190 Boulevard Crémazie Est, Montréal, QC H2P 1E2 Canada; 3 School of Nursing 3H48C, McMaster University, 1280 Main Street West, Hamilton, ON L8S 4K1 Canada; 4 École de réadaptation, Université Sherbrooke, 3001, 12e Avenue Nord, Sherbrooke, QC J1H 5N4 Canada; 5 Institute of Health Policy, Management and Evaluation, University of Toronto, Health Sciences Building, 155 College Street, Suite 425, Toronto, ON M5T 3M6 Canada; 6 Nuffield Department of Primary Care Health Sciences, Radcliffe Observatory Quarter, Woodstock Road, Oxford, OX2 6GG UK; 7 Department of Anthropology, Mcgill University, 7th Floor Leacock Building, 855 Sherbrooke Street West, Montreal, QC H3A 2T7 Canada; 8 InterActions, centre de recherche et de partage des savoirs, 11 822, avenue du Bois-de-Boulogne, Montréal, QC H3M 2X7 Canada; 9 Department of Community Health and Epidemiology, Centre for Clinical Research, Dalhousie University, Room 425, 5790 University Ave, Halifax, NS B3H 1V7 Canada; 10 School of Nursing, Dalhousie University, Room G26, Forrest Bldg., PO Box 15000, 5869 University Ave, Halifax, NS B3H 4R2 Canada; 11 School of Public Health, 3-300 Edmonton Clinic Health Academy, University of Alberta, 11405 – 87 Ave, Edmonton, AB T6G 1C9 Canada; 12 UBC Centre for Health Services and Policy Research, Vancouver Campus, 201-2206 East Mall, Vancouver, BC V6T 1Z3 Canada; 13 Department of Community Health Sciences, Max Rady College of Medicine, University of Manitoba, Room S113-50 Bannatyne Ave, Winnipeg, MB R3E 0W3 Canada; 14 Dalla Lana School of Public Health, University of Toronto, Health Sciences Building 155 College St., Room 540, Toronto, ON M5T 3M7 Canada; 15 Department of Preventive Medicine, School of Medicine, University of Southern California, 2001 N. Soto Ave, Room 302w, Los Angeles, CA 90034 Canada; 16 School of Public Administration, University of Victoria, PO Box 1700, STN CSC, Victoria, BC V8W 2Y2 Canada; 17 Canadian Nurses Association, 50 Driveway, Ottawa, ON K2P 1E2 Canada

**Keywords:** Health-relevant policies, Heath policy, Knowledge exchange, Policy-making, Social network analysis

## Abstract

**Background:**

Health systems worldwide struggle to identify, adopt, and implement in a timely and system-wide manner the best—evidence-informed—policy-level practices. Yet, there is still only limited evidence about individual and institutional best practices for fostering the use of scientific evidence in policy-making processes The present project is the first national-level attempt to (1) map and structurally analyze—quantitatively—health-relevant policy-making networks that connect evidence production, synthesis, interpretation, and use; (2) qualitatively investigate the interaction patterns of a subsample of actors with high centrality metrics within these networks to develop an in-depth understanding of evidence circulation processes; and (3) combine these findings in order to assess a policy network’s “absorptive capacity” regarding scientific evidence and integrate them into a conceptually sound and empirically grounded framework.

**Methods:**

The project is divided into two research components. The first component is based on quantitative analysis of ties (relationships) that link nodes (participants) in a network. Network data will be collected through a multi-step snowball sampling strategy. Data will be analyzed structurally using social network mapping and analysis methods. The second component is based on qualitative interviews with a subsample of the Web survey participants having central, bridging, or atypical positions in the network. Interviews will focus on the process through which evidence circulates and enters practice. Results from both components will then be integrated through an assessment of the network’s and subnetwork’s effectiveness in identifying, capturing, interpreting, sharing, reframing, and recodifying scientific evidence in policy-making processes.

**Discussion:**

Knowledge developed from this project has the potential both to strengthen the scientific understanding of how policy-level knowledge transfer and exchange functions and to provide significantly improved advice on how to ensure evidence plays a more prominent role in public policies.

## Background

New conceptual and methodological developments in the broad field of knowledge transfer and exchange suggest significant improvement in policies and practices could be achieved by shifting the focus of analysis from discrete interventions to broader information exchange networks. This proposal aims to map and analyze health-relevant information exchange networks at the national level in Canada. It will lead to concrete best practice recommendations with the potential to improve the integration of scientific evidence into health-relevant policies and practices and ultimately have a positive impact on the health of Canadians.

### Significance and objectives of the research

Health systems worldwide struggle to identify, adopt, and implement in a timely and system-wide manner the best—evidence-informed—policies and practices. This struggle, in turn, has significant implications for resources and population health [1–3].

A large body of scholarship has focused on developing interventions to strengthen the influence of scientific evidence on decisions and policies. However, despite significant energy and investments, efforts to do so have proved trickier than initially anticipated [3, 4]. The complexity of policy-level^1^ knowledge transfer and exchange (KTE) interventions has thwarted attempts to produce strong instrumental evidence on the “how-to” [3, 5, 6]. Part of the problem is rooted in the fact that much of the KTE literature focuses on discrete “interventions.” In practice, policy-making processes take place in complex networks where actors are interdependent and where KTE is neither linear nor discrete. Further inquiry into the composition and functioning of the channels through which information informs practices and decisions is crucial to identify best practices for fostering use of scientific evidence [3, 7–16].

This project’s main objective is thus to understand how scientific evidence interconnects with health-relevant policy-making processes. Operationally, this will be achieved by focusing on the composition and structure of complex policy networks and then analyzing the processes of information circulation and absorption within these networks. We define health-relevant policies as encompassing both healthcare policies (i.e., policies about healthcare services financing or delivery) and healthy public policies (i.e., intersectoral policies with significant implications for population health and health equity).

More specifically, this project adopts a sequential mixed-methods approach, structured in two components with three specific objectives:

1. To map and structurally analyze—quantitatively—health-relevant policy-making networks that connect evidence production, synthesis, interpretation, and use (component A).
2. To select a subsample of actors with high centrality metrics or interesting structural positions within these networks and qualitatively investigate their communication and interaction patterns, to develop an in-depth understanding of evidence circulation processes and related strategies (component B).
3. To combine these findings in order to assess a policy network’s absorptive capacity regarding scientific evidence and to integrate them into a conceptually sound and empirically grounded framework (integration of components A and B).

### Conceptual framework

Conceptually, this project is at the intersection of three fields of research. The first—usually referred to in Canada^2^ as KTE—is focused on analysis and improvement of the bidirectional linkages between scientific evidence production and policy or practice. The second field—policy-making analysis—is anchored in political science and public administration and is focused on understanding structures and processes that influence public policy development, adoption, and implementation, conceptualized as dependent variables. The third field—network analysis—is transdisciplinary, often very methodologically driven, and focused on network structures as independent variables explaining a diverse range of phenomena.

Although there is a considerable body of literature in each of these fields on the influence and use of scientific evidence in policy formulation and making, their intersection has only been partially explored (e.g., reviews about KTE and networks [17]; policy-making and networks [18–20]; KTE and policy-making [4, 5]). Few, if any, studies have tapped into cross-learning from all three. However, developments in those three fields support a redefinition of how policy-related KTE interventions should be conceptualized. More realistic conceptualizations should take into account that information exchanges in policy-making processes involve heterogeneous actors (beyond researchers, civil servants, and managers) and are both collective (rather than involving sovereign autonomous decision-makers) and systemic (rather than step-based, as in linear or circular models).

We broadly define policy networks here as the structures and processes of interaction among individuals and organizations engaged in a policy field [21–23]. This definition highlights the heterogeneity of policy actors and arenas. Policy networks are not confined to government authorities and formal decision-makers but also include all other actors who work on policies or seek to influence, transform, or shape policies, such as non-governmental organizations, activists, industries, interest groups, or the media [24, 25]. In this perspective, networks do not have formal boundaries; they are informal, self-organizing, and in continual transformation [26–29].

By collective, we mean that policy processes occur in systems with a high level of interdependency and interconnectedness among participants [17, 18, 30]. Interdependency here refers to the fact that usually none of the participants dispose of enough autonomy or power to translate the information into practices on their own [31–39]. In such contexts, individuals are embedded within systemic relations, where knowledge use depends on processes such as sense-making [40–42], coalition-building [8, 43, 44], developing trustworthiness [45, 46], and rhetoric and persuasion [42, 47–49].

Such a view calls for a broader conceptualization of policy-making, in both the processes and the actors involved. Although the fields of policy analysis and KTE have been very much influenced by the concepts of “decision” and “decision-making,” operationalizing those concepts in collective systems [37, 50] can be highly problematic [51–53]. In contrast, policy processes are systemic, in that they involve a slowly evolving set of participants interacting over long periods [31, 39, 50, 54–60]. Discrete decisions or events are never the end of an identifiable process, but rather steps in a broader game [26, 61–64].

The sophistication of the policy-making concept summarized above highlights the importance of understanding how the structure formed by policy actors’ interactions with each other influences the circulation and absorption of scientific evidence. Converging evidence suggests the connection between scientific results and policy-makers’ practices is strengthened in policy networks/subnetworks in which scientific evidence “sources” or “producers” occupy, on average, a more central position. Based on social network analysis methods and theories, there is strong conceptual [65–69] and empirical [17, 18, 21, 56, 70–75] evidence to support the hypothesis that actors in bridging positions and/or with high centrality wield more influence. Sandström and Carlsson’s work [18, 30, 55], for example, demonstrates that subnetworks with high actor heterogeneity, high density, and high whole-network centrality are more desirable for effective KTE.

Accordingly, our aim in this proposal is to shift the focus of KTE analysis to (1) the structure of the interconnections between actors and (2) behaviors and communication processes (ties) as core determinants of the influence of scientific evidence in policy-making processes.

Such a focus prompts a shift in effect attribution. Most KTE literature is based on causal attribution models, in which intervention effectiveness is seen as attributable to characteristics of the strategy, users, or producers. However, if the structure of interconnections between actors is indeed a core determinant of KTE effectiveness, those attribution models are inappropriate [17, 30, 75]. What becomes crucial is understanding the network structure and its functioning. As described in more detail below, this project combines quantitative structural analysis of actors’ positions with qualitative analysis of their behaviors and communication processes from a network perspective.

## Methods

**Fig. 1:**
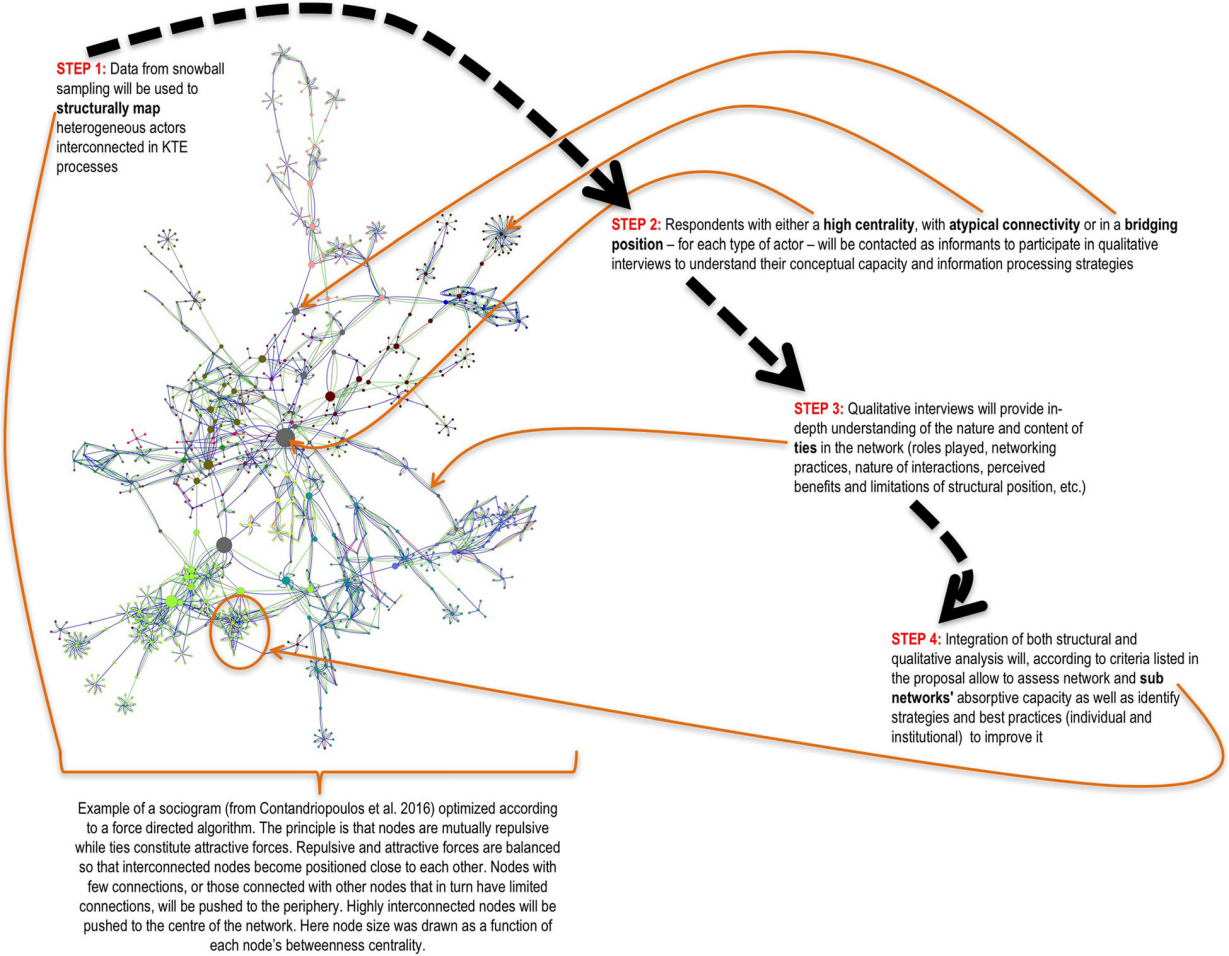
Example of a sociogram

**Fig. 2:**
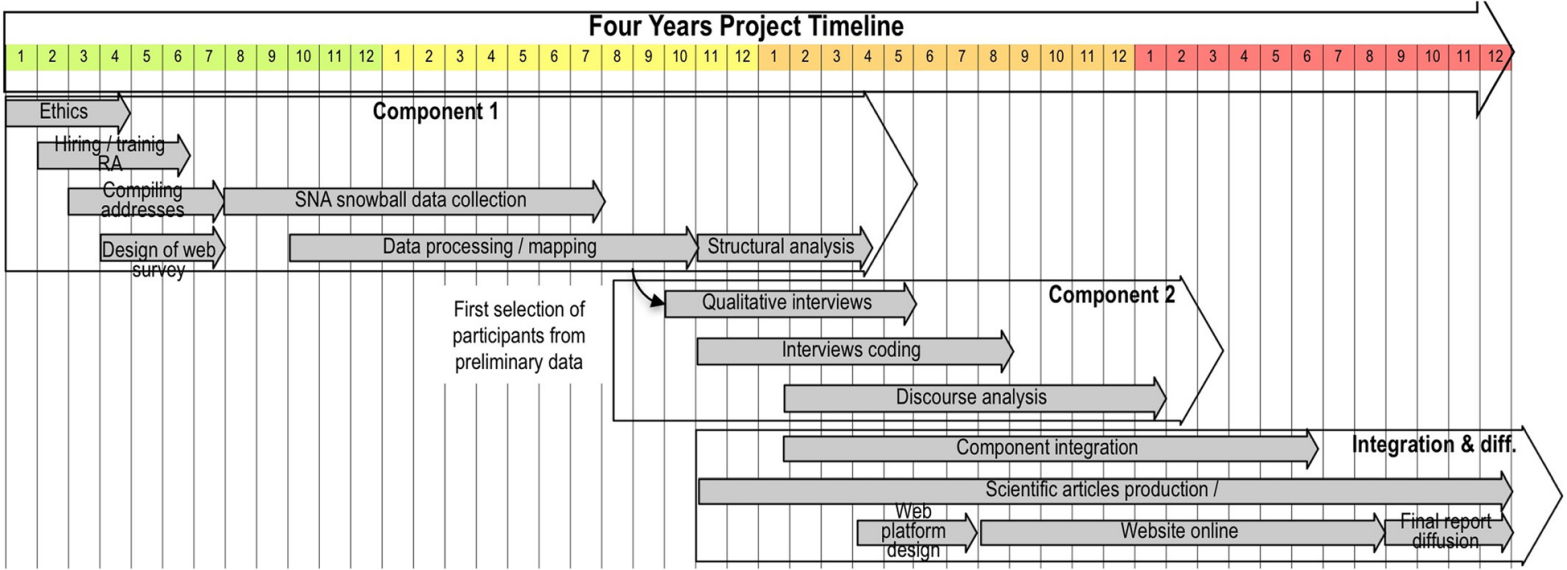
Four-year project timeline

As highlighted in the previous section, understanding policy-related KTE processes implies shifting the focus of analysis in two ways: first, by relying on a more realistic conceptualization in which KTE is seen as the product of collective and systemic exchange networks of heterogeneous actors, and second, by combining structural network analysis with information about actors’ behaviors, resources, and skills in communication processes and actors’ perceptions of their capacity to act upon/influence policy-making processes [17]. For this reason, the present project will use a mixed-methods approach [76–78] with two components: (A) mapping and structurally analyzing Canadian health-relevant policy networks through multi-step snowball sampling and (B) qualitatively analyzing the processes through which scientific evidence circulates, based on interviews with a purposeful sample of significant actors in the network. Results from both components will then be integrated into a unified, conceptually sound, and empirically grounded framework (see Fig. 1 for a visual summary of the research design and Fig. 2 for a visual summary of the timeline of research activities).

### Component A: network mapping and structural analysis

This component is aimed at identifying the actors involved in health-relevant policy networks in Canada and analyzing their structural position within these networks (objective 1). This method is based on quantitative analysis of relations (ties) that link nodes (here, individual actors) in a network.

#### Data collection and research participants

Network data will be collected through a multi-step snowball sampling strategy. The first challenge in implementing such an approach is to set conceptually sound and operationally manageable boundaries for the network being sampled. For this, we will apply two typologies. The first is a typology of actors and spheres of action based on the policy network literature [45, 79]:

- *Political sphere*: Elected decision-makers at the federal, provincial, and municipal levels;
- *Public administration*: Civil servants at the federal, provincial, and para-governmental institutional levels;
- *Academia*: Researchers/professors in universities and other, mostly publicly funded, research institutions;
- *Media*: Journalists and other news producers in broadcast, print, and electronic media;
- *Civil society*: Interest groups, advocacy coalitions, unions, nongovernmental organizations, foundations, transnational agencies, and professional associations;
- *Private sector*: Private corporations and industries.

The second typology is focused on the operational definition of what we have described as health-relevant policies. Healthcare policies and healthy public policies include a wide range of complex interventions which often share few similarities aside from their ultimate goal of positively impacting the health of individuals and populations [6]. To structure the delimitation of the field, we integrated and adapted the OECD typology of health policies with the WHO typology of healthy policies [80–82]. The end result is a heuristic classification with no pretension of exhaustively listing all subfields. Its role is to help in the inductive identification of informants in each sphere’s fields and subfields.

These two typologies are the starting point of our snowball strategy. They will be used to build and structure an initial list of actors and organizations considered to be involved in shaping or trying to influence health-relevant policies in Canada. Sources used to compile an initial list of names and contact information in each of these spheres, per province, will consist of publicly available directories and institutional websites, social media platforms, and reference lists provided by each of the team’s co-applicants and collaborators based on each Canadian region. The health-relevant policy fields and subfields provided in Table 1 will be used as a structure both to generate keywords for online searches and to define the boundaries of the data collection effort. Pilot testing of the approach suggests we will be able to compile initial lists of hundreds of contacts per category. We aim to launch the approach with between 2000 and 5000 initial contacts.

**Table 1:** Health-relevant policy fields and subfields

Healthy public policies	
Policies across spheres that explicitly take into account their implications for population health and health equity	
Prevention and health promotion	- Food and nutrition - Alcohol/tobacco/addiction - Chronic diseases and long-term care - Disease surveillance (communicable and non-communicable)
Social, economic, environmental determinants, and health equity	- Housing - Transport - Education - Income/fiscal policies - Employment - Social assistance
Healthcare policies	
Policies about healthcare services financing or delivery	
Health financing and funding	- Universal health coverage - Payment and insurance systems - Health systems characteristics - Equity and access to health services and products - Funding policy - Hospital funding
Health system service delivery	- Quality of care - Coordination of care - Primary healthcare - Community care - Home care - Hospital services
Health data governance and infrastructure	- Data governance: privacy, monitoring, and research - Strengthening health information - Infrastructure for healthcare quality governance - Data-driven innovation: big data for growth and well-being

Potential respondents will be contacted by mail, email, and phone (details below) and invited to complete a short bilingual online survey structured around four themes: (1) provide informed consent, (2) answer a few descriptive questions on personal characteristics (specifically professional occupation [the sampling category]; perceived KTE role(s) [along the producer/broker/user division]; institutional affiliation; hierarchical position held in the institution; and geographic location), (3) identify the health-relevant policies in which they are involved (closed questions built from Table 1 as a starting point and finalized after pilot testing; respondents will also be able to identify other policy themes on which they are working by selecting the option “other”), and (4) nominate up to ten people with whom they are in contact regarding their involvement in policy-relevant processes. Previous work by the team with this method suggests saturation occurs before ten responses [83].

Participant eligibility will be based on self-perception, in that any individuals who consider themselves actively involved in health-relevant policy processes at the institutional, provincial, or federal levels will be eligible. For every element in the survey, an operational definition will be displayed onscreen using a mouse-over function (e.g., for question 4: “Being in contact with is here defined as a regular or irregular form of personal communication, either face-to-face or via email, phone, or social media”). The survey is expected to take around 5 min to complete. We will use the Polinode platform (www.polinode.com), an online tool specializing in relationship-based surveys and network analysis.

Respondents identified through this peer-nomination process will, in turn, be invited to fill out the survey and identify their own network of contacts. This multi-step snowballing process is a common way to identify actors in unbounded networks, such as policy networks, and has been used in other studies to identify policy-makers and/or influential actors in policy-making [21, 79]. This method reduces initial sampling bias, since single-step sampling is generally restricted to actors assumed to be the most active in formal settings and/or publicly visible. By including other meaningful contacts who are not necessarily the most visible or expected actors, the recursive name generation of contacts-of-contacts expands the network and captures its heterogeneity.

The main challenge of this data collection process is to obtain a satisfactory response rate. Following Dillman’s tailored design best practices [84], we will use a sequential multiple contact strategy to stimulate participation. Potential participants will receive both personalized email and mail invitations, with a token incentive [85]. Mail and email reminders will be sent 2 weeks later, followed by a phone call to nonrespondents a week later during which they will be given the possibility to respond to the survey by phone. The project involves partners, co-investigators, and collaborators with extensive contact lists in all provinces. These contacts will be used to personalize mail and email invitations in order to increase the response rate.

The snowball data collection period will run for 1 year of active follow-up, with the objective of obtaining 20 to 50% response rates in each policy actor sphere. As there are no reliable estimates of the number of potential participants in each of these categories, we will use the Cormack-Jolly-Seber “capture–mark–recapture” model [86], as implemented in the Program MARK software (http://www.phidot.org/software/mark/background/), to calculate the estimated whole population of actors in each subgroup. Each person identified as pertaining to one of the categories of actors we aim to sample will be considered “captured.” Each person already on our list whose name emerges through the snowball name-generator question will be considered “recaptured.” Using this method, total population estimates per policy actor sphere will be computed during data collection. At the end of data collection, the model will also provide reliable estimates of the proportion of the overall network for which we have data. This is a crucial issue for social network analysis (SNA) ego-based snowball sampling, as the networks obtained are always only a bounded extraction from a, practically, limitless network [21, 87, 88].

#### Data analysis and interpretation

The data obtained through the multi-step snowball sampling (names of participants and of the people with whom they are in contact) will be transposed into a symmetric matrix where each row/column corresponds to a node (actor) and where values correspond to ties (relations between actors). From such a matrix, it is possible to produce a network map (sociogram) and to compute network-, cluster-, and node-level metrics [68, 87–92]. To do this, the data will be imported into and analyzed with UCINET 6 software. Sociogram visual optimization will be done on Cytoscape 3.3.0 software through force-directed algorithms (see Fig. 1). The data will then be analyzed structurally using SNA and graph theory [65, 68, 87–92]. To identify central actors, actors in bridging positions, and actors with atypical connectivity [65, 68, 91] in the networks, node (degree, closeness, betweenness, and eigenvector centralities) and network (density, clustering, and structural holes) structural metrics will be computed. Actors’ personal characteristics will be plotted on the graph to identify shared attribute patterns (homophily) [93]. We will also use multiple regression models (in SPSS 23.0) to test the statistical association between actors’ characteristics and structural node metrics. Finally, community detection algorithms will be used to understand underlying clustering factors. Conceptually coherent clusters (i.e., based on node homophily, policy issues, or geographic proximity) will be identified and treated as policy subnetworks. We will also measure the interconnectedness of these health-relevant policy subnetworks.

Results from this structural analysis will be interpreted at three levels. First, we will assess the whole-network connectivity of actors labeled as scientific evidence “sources/producers” in health-relevant policy networks in Canada. Then, we will compare policy subnetworks based on the assessment criterion that policy subnetworks in which scientific evidence sources are, on average, more central are more desirable. Finally, we will compare the KTE potential of subnetworks, based on Sandström and Carlsson’s work [18, 30, 55] showing that subnetworks with high actor heterogeneity, high density, and high whole-network centrality are more desirable.

### Component B: qualitative analysis of communication processes and perceived influence

As stated earlier, structural position alone does not explain how knowledge can be efficiently circulated and transferred in health policy networks; factors such as conceptual capacity and political clout must also be taken into consideration. For example, an actor may be in a structural position that enhances his/her exposure to relevant information but be ill-equipped, in practical terms, to make sense of this information [5, 94, 95] or to use it to influence others [5, 14, 47, 96]. Conversely, an actor could have low structural connectivity, and thus limited exposure to relevant information, but still have significant conceptual capacity. We will rely on the concept of *absorptive capacity* to bridge these two notions of structural position and conceptual capacity. An actor with high absorptive capacity [95, 97] has both the opportunities (high structural exposure to new knowledge [18, 56, 67, 68, 71, 73]) and the means (prior knowledge and practical capacity [8, 23, 41, 46, 94]) to foster use.

To understand actors’ behaviors and information processing strategies through which structural network connectivity are operationalized, we will conduct qualitative semi-structured interviews with a purposeful subsample of the Web survey participants. This will allow us to understand both how actors end up in a particular structural position and whether actors’ views on their capacity to access evidence, transfer information, and ultimately influence policy-making correspond to the theoretical advantages that specific network positions and structures are assumed to provide (e.g., central and bridging positions).

#### Data collection

Informant selection will be based on the actor-level and cluster-level structural metrics obtained from structural analysis (component A). For each actor type and subnetwork, we will invite a combination of actors (maximum-variation sampling strategy) with high prestige (degree centrality and eigenvector centrality), high bridging (betweenness centrality and structural-hole position), and atypical connectivity [65, 68, 91] in the whole network and within subgroups/clusters to participate in in-depth semi-structured interviews of approximately 60 min. Clusters identified through component A—including data about each node’s real name/organizational affiliation—will be discussed with all co-investigators and collaborators to look for ideological or interest-based clustering effect [23, 98, 99]. We plan to conduct between 40 and 60 such interviews. As informants will be spread throughout Canada, interviews will be conducted either by phone or Skype depending on informants’ preferences. Interviews will be conducted in the informant’s preferred language and will be recorded (with informed consent), transcribed, proofread, and imported into ATLAS.ti 7 qualitative data analysis software for coding and analysis.

For each participant, the main themes covered will be

- Themes/issues/policies in which he/she is involved in the network.
- Role played in the network and modes through which this role is enacted (public media appearances, advocacy, participation in public or stakeholder forums, membership in government committees or advisory groups, provision of direct advice or assistance in policy-making processes, collaborative research, and/or personal communications with official policy- and decision-makers).
- Networking motives and practices (how did the participant come in contact with the people listed in the name generator survey, how does the participant create new contacts, for what reasons, what are the participant’s needs/expectations when seeking new contacts).
- Advantages/limitations of network positions (are current network relations useful, which are most useful and why, level of difficulty in establishing/finding necessary contacts).
- Perceived influence in the network (personal assessment and opinion on which type of actor has more influence on policy-making and why, modes of participation in the network that seem more efficient for using and disseminating scientific evidence, and external factors that facilitate or limit individual capacity to play an effective role in the network, e.g., organizational affiliation, professional occupation, and hierarchical position).

#### Data analysis and interpretation

Transcript coding will be based on systematic identification of recurring themes [100, 101]. Codes will be developed inductively as the analysis unfolds, but the starting point will be anchored in the complementary dimensions put forward in the works of C Phelps, et al. [17], Sandström and Carlsson [18], and Sabatier and Jenkins-Smith [60]. Discourse analysis approaches [100–102] will be used. Each coded interview will first be analyzed independently and then transversally, by comparing similarities and differences between policy subnetworks and actors’ characteristics. We will use investigator triangulation (*n* = 2) to ensure coding reliability [103, 104]. Codebook definitions and analysis will be scrutinized and discussed in group meetings with all co-investigators and research assistants. The research team has extensive experience successfully using similar qualitative data analysis.

## Discussion

### Results integration and impact

As stated in previous sections, the project’s main objective is to provide a conceptually sound and empirically grounded understanding of the way by which scientific evidence interconnects with decision-making at policy levels in Canada. To achieve this (ambitious) goal, the project relies on a mixed-method design with two components. Conceptually, the integration of both components’ results will involve extending the notion of absorptive capacity to the subnetwork level. Absorptive capacity is conceived here as both a property of subnetworks’ structural properties and the optimization of actors’ communicative strategies within a given structural arrangement. This extension tallies with existing evidence on collective effects in innovation adoption [75, 97] and knowledge use [51, 105]. The results will shed light on the relative structural position of individuals and institutions within subnetworks, the communications strategies they use, and the factors (interests and ideologies) that explain them.

This project is based on cutting-edge, interdisciplinary conceptual developments [17, 18, 20, 24, 55, 79] and innovative large-scale data collection methods. Conceptually, it addresses some of the main challenges that have vexed collective-level knowledge transfer and exchange (KTE) literature. Knowledge developed from this project has the potential both to strengthen the scientific understanding of how collective KTE functions and to generate significantly improved practical advice on how to strengthen the role of evidence in organizational practices and public policies. Ultimately, this can lead to more effective integration of scientific evidence into practices and to decisions that can have a beneficial impact on the health of Canadians.

### Dissemination of results

The KTE plan for this project is threefold. First, this project is conceptually innovative and relies on a data collection approach that has, to the best of our knowledge, never been used at the national level. We believe the results will lead to high-impact scientific articles with potential to influence the field.

Second, the project involves three key partner organizations that will be actively involved in both the use of the project’s results (user role) and their dissemination to other potential users (vector role): the National Collaborating Centre for Healthy Public Policy (NCCHPP), the Canadian Nurses Association (CNA), and the Ontario Tobacco Research Unit (OTRU).

Partnerships with our three main partner organizations will be paramount in helping the research team contextualize the findings, adapt them to the needs of users involved in organizational decision-making and policy-making, and disseminate them to relevant stakeholders. Adapted and summarized findings will be formatted both in a 1:3:25 report and as an interactive website. Beyond the three core partners identified above, we will also mobilize collaborations with the Evidence Network and the six National Collaborating Centres for Public Health to disseminate results to potential users. In the same way, our team is truly pan-Canadian in scope, and co-investigators’ and collaborators’ connections with other significant actors in Canada will be used to foster more such collaborations as the project unfolds.

The third element of the plan is that a fundamental product of this project will be the development of a nominal map of thousands of actors involved in health-relevant policies across Canada. This map in itself will represent a KTE instrument of remarkable possibilities. All individuals identified through the project’s data collection efforts will receive an email link to the report and interactive website results. The network map will be uploaded to the website, and participants will be able to log in and locate their exact position (node), interconnections in the network, and personal centrality metrics. This sharing of results with participants also has the potential to play an important role in disseminating results. Active traffic monitoring strategies and social media will also be used to foster access. Team expertise and resources will also be mobilized for that purpose, especially to attract mass media attention as a part of the end-of-grant KTE plan.

### Expertise, experience, and resources

This project is ambitiously integrative at both the conceptual and methodological levels. Essential to its success is a team of researchers with complementary individual expertise and, collectively, a truly outstanding track record. The pan-Canadian composition of the team is also a key strength, as team members’ knowledge of health-relevant policies and policy actors in their respective provinces will be essential for designing research tools, identifying and reaching out to participants, disseminating findings and results, and adapting them to local needs.

## Abbreviations

CNA: Canadian Nurses Association
KTE: Knowledge transfer and exchange
NCCHPP: National Collaborating Centre for Healthy Public Policy
OECD: Organisation for Economic Co-operation and Development
OTRU: Ontario Tobacco Research Unit
SNA: Social network analysis
WHO: World Health Organization

## Footnotes

1 We define policy-level interventions broadly to include federal, provincial, and para-governmental institutional-level interventions. A more operational definition is provided in the next pages.

2 The phenomenon we label knowledge transfer and exchange here is described under a variety of terms depending on the country and discipline. Common terms include knowledge translation, knowledge exchange, and implementation research.
